# Prevalence of Hepatitis C Virus Seropositivity and Its Impact on Coronary Artery Disease among Egyptian Patients Referred for Coronary Angiography

**DOI:** 10.1155/2016/1623197

**Published:** 2016-11-02

**Authors:** Ragab Abd El Salam, Baher Nabil, Marawan Saber, Hany A. AbdelWahab, Tamer Saber

**Affiliations:** ^1^Cardiology Department, Faculty of Medicine, Zagazig University, Zagazig, Egypt; ^2^Internal Medicine Department, Faculty of Medicine, Zagazig University, Zagazig, Egypt

## Abstract

*Background*. We tested the prevalence and impact of HCV seropositivity among Egyptian patients referred for coronary angiography.* Subjects and Methods*. This cross-sectional study was conducted in Zagazig University hospitals including 509 patients scheduled for elective coronary angiography between June 2013 and June 2014. By taking full history on admission, laboratory workup including HCV Ab, echocardiography study, and coronary angiography, we calculated the mean number of coronary artery lesions and the mean number of affected coronary artery vessels for all patients. The severity of the coronary lesions was estimated using the Gensini score.* Results*. HCV seropositive patients referred for coronary angiography were about 30.3% (which is greater than the prevalence of HCV seropositivity among general population in Egypt), patients proved to have CAD who are HCV antibody positive had more severe coronary lesions than in seronegative one (*p* < 0.05), and patients proved to have CAD who are HCV antibody positive had comparable prevalence of cardiovascular risk factors as seronegative patients except for diabetes and hypertension which are more prevalent in seronegative patients (*p* < 0.05).* Conclusion*. Prevalence of HCV antibody positive patients referred for coronary angiography was about 30.3%, and CAD patients who are HCV antibody positive had more severe coronary lesions and less prevalence of diabetes and hypertension than HCV antibody negative.

## 1. Introduction

The World Health Organization (WHO) has declared hepatitis C virus a global health problem, with approximately more than 185 million people around the world having been infected with HCV [1]. In Egypt, HCV infection was estimated most recently at 14.7% among subjects 15–59 years old and 18% among rural residents, according to the 2008 Egypt Demographic and Health Survey conducted on behalf of the Egyptian Ministry of Health [2].

Coronary artery disease (CAD) continues to be the main cause of death and a major cause of morbidity and loss of quality of life. CAD is a leading public health problem accounting for a significant proportion of total societal costs [3].

Atherosclerosis, either subclinical or manifest, is a chronic inflammatory disease. The chief clinical manifestations are CAD, stroke, and ischemic limb. The possible role of an infectious agent in the development of experimental atherosclerosis in rodents was first reported more than 120 years ago [4] and this concept has gained new life in recent years [5]. In fact, chronic HCV infection causes hepatic and systemic inflammation* via* increased levels of proatherogenic chemokines and cytokines [6]. In addition, it has been demonstrated that HCV colonizes and replicates within carotid plaques likely causing vascular inflammation [7]. Earlier studies conducted in the general population showed that HCV markers were independently associated with atherosclerosis [8]. Subsequent research, however, yielded conflicting results [9–12].

*Aim of the Work*. The aim of this study was to measure the prevalence of HCV seropositivity among Egyptian patients referred for coronary angiography and to assess the impact of HCV seropositivity on coronary artery disease in the patients referred for coronary angiography.

## 2. Patients and Methods

### 2.1. Study Design and Population

This cross-sectional study was conducted in Zagazig University hospitals. We included patients scheduled for elective coronary angiography within a time period of twelve months from June 2013 to June 2014.

*Exclusion Criteria*. Previous percutaneous coronary intervention (PCI) with coronary artery stenting or coronary angioplasty, previous coronary artery bypass grafting (CABG), contraindications to dye as dye allergy, and renal failure are the exclusion criteria.

Clinical and demographic data were collected for all patients at time of hospital admission: age, gender, major cardiovascular risk factors including diabetes mellitus, hypertension, dyslipidemia, smoking, and family history of CAD. The indication for coronary angiography and history of prior cerebrovascular stroke (CVS), peripheral vascular disease (PVD), prior myocardial infarction (MI), congestive heart failure (CHF), chronic obstructive pulmonary disease (COPD), and obstructive sleep apnea (OSA) were also collected.

*Laboratory Workup*. Laboratory workup was fasting plasma glucose (FPG), fasting lipid profile (total cholesterol, LDL, HDL, and TGs), serum creatinine, and hepatitis C virus antibodies using the third-generation ELISA method [13].

### 2.2. Echocardiography Study

Left ventricular end systolic dimensions and end diastolic dimensions, left ventricular ejection fraction, and regional wall motion score index (WMSI) were used.

### 2.3. Coronary Angiography

All patients had undergone coronary angiography. Digital coronary angiograms were analyzed offline with an automated edge detection system (Philips Integris 5000, Netherland) by using the dye-filled guiding catheter as a reference, and coronary angiography was performed in multiple projections for adequate analysis of target lesions.

Angiographic results were interpreted by two angiographers who were blinded to clinical or demographic data. Coronary angiography was utilized to identify plaques (at least 20%) indicating the presence of atherosclerosis.

We calculated the mean number of coronary artery lesions and the mean number of affected coronary artery vessels for all patients. We also calculated the percentage of patients who had coronary artery chronic total occlusion.

### 2.4. Calculation of Gensini Score

The severity of the coronary lesions was estimated using the Gensini score [14], based on the degree of luminal obstruction which may involve either concentric or eccentric lesions. The score for each segment was multiplied by a weighing factor reflecting the importance of that particular coronary segment

(1) Gensini score=∑points for each segment×weighing factor.

The points were assigned starting at 25% occlusion with values doubled for each increasing level of occlusion [14, 15].

### 2.5. Statistical Analysis

Data were analyzed with SPSS software version 16.0 for Windows (SPSS Inc., 2007). Continuous data are presented as means ± SD. Differences in continuous variables between groups were determined by Student's* t*-test (normal) or Mann–Whitney* U *test (nonnormal). Categorical variables are presented as percentages and compared with chi-square test or Fisher's exact test. The Pearson correlation was calculated to evaluate the association between 2 continuous variables.

## 3. Result

The study included 509 patients, classified into group (1) which included patients proved to have CAD with coronary angiography and group (2) which included patients with normal coronary angiography.

### 3.1. Demographic Characteristics of the CAD Positive and CAD Negative Groups

Both CAD positive group and CAD negative group had comparable age, gender distribution, and family history.

There was statistically significant difference between both groups regarding hypertension, diabetes, smoking, dyslipidemia, and HCV prevalence, where all were more prevalent in CAD positive group as shown in Table 1.

Group (1) (CAD positive) was further subdivided into group (1A) which included HCV antibody positive patients and group (1B) which included HCV antibody negative patients.

Both group 1A (HCV Ab positive) and group 1B (HCV Ab negative) had comparable age, gender distribution, dyslipidemia, smoking, family history, and clinical presentation. However, there was statistically significant difference between both groups regarding hypertension and diabetes, where hypertension and diabetes were more prevalent in HCV Ab negative group (Table 2).

Both HCV Ab positive group and HCV Ab negative group had comparable clinical presentation.

**Figure 1:**
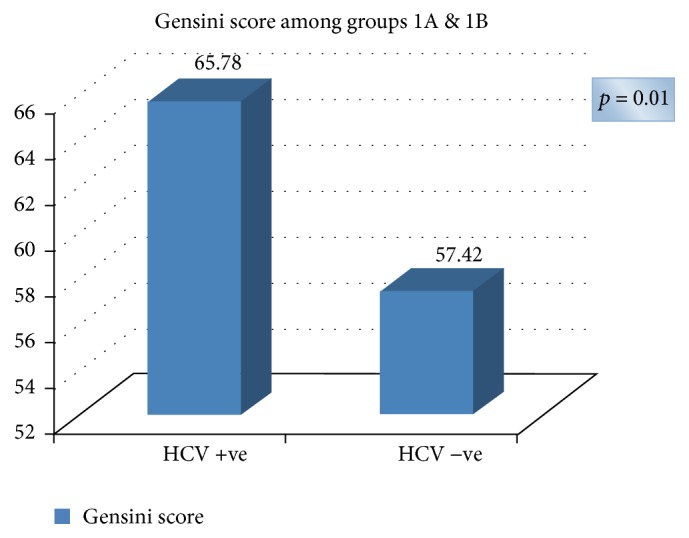
Gensini score among HCV Ab +ve and −ve groups.

And ejection fraction distribution is as shown in Table 3. However, there was statistically significant difference between both groups regarding Gensini score, where Gensini score was higher among HCV Ab positive group as shown in Table 3 and Figure 1.

## 4. Discussion

Several studies have suggested that some infectious agents may cause cellular and molecular changes that contribute to the pathogenesis of atherosclerosis [16]. The data obtained indicate the identification of viral genomes in the atherosclerotic plaques and also proatherogenic effects of viral infection in cells relevant to atherogenesis (smooth muscle cells, monocyte macrophages, T cells, and endothelial cells) [16].

HCV infection is increasingly identified as a potential atherogenic condition. In fact, chronic HCV infection causes hepatic and systemic inflammation [6] via increased levels of proatherogenic chemokines and cytokines [6] and hepatic steatosis, a distinguishing feature of this infection [17]. Also, it has been demonstrated that HCV colonizes and replicates within carotid plaques likely causing vascular inflammation [7]. Earlier studies conducted in the general population showed that HCV markers were independently associated with atherosclerosis [18]. Subsequent research, however, yielded conflicting results, some studies confirming [9, 19] and others denying such an association [10, 20]. However, more data have shown excess cardiovascular mortality during the course of chronic HCV infection [11, 12]. Given the high prevalence of HCV infection on a worldwide basis and in Egypt and the primacy of cardiovascular diseases among the causes of mortality, the role of HCV as a cardiovascular risk factor needs to be analyzed in-depth.

In the present study, we evaluated the prevalence of HCV seropositivity and its impact on coronary artery disease among Egyptian patients referred for coronary angiography in our cath lab in the Cardiology Department of Zagazig University hospitals and tried to test the association of HCV infection with other risk factors of cardiovascular disease and with the pattern and severity of coronary artery involvement using the angiographic scoring system, Gensini score.

Our study showed a prevalence of HCV seropositivity among the patients referred for coronary angiography about 30.3% which is greater than the prevalence of HCV seropositivity among general population in Egypt which was estimated to be about 14.7% among subjects 15–59 years old, according to the 2008 Egypt Demographic and Health Survey conducted on behalf of the Egyptian Ministry of Health [2].

The prevalence of HCV Ab positivity was significantly higher in the group of patients proved angiographically to have CAD than the group of patients with normal coronary angiography (8.75 ± 1.69 versus 6.01 ± 1.80, *p* < 0.001) (*p* < 0.05).

This study showed that patients with CAD who are HCV Ab positive had a comparable prevalence of cardiovascular risk factors as seronegative patients proved to have CAD except for diabetes and hypertension which are more prevalent in seronegative patients (*p* < 0.05). Despite that, the severity of coronary artery lesions which was assessed by Gensini score was significantly higher in HCV Ab positive group (*p* < 0.05).

This was similar to the study of Butt et al. [21] who conducted the largest epidemiological study (82 083 HCV-infected and 89 582 HCV-uninfected subjects) in United States veterans over a 5-year period. The data showed that HCV-infected subjects had a significantly higher prevalence of cardiac diseases (myocardial infarction, congestive heart failure, coronary artery bypass grafting, or coronary angioplasty) despite being younger and having a more favorable cardiometabolic risk profile [21].

Also our study findings were consistent with a case-control study, which included 686 patients; Vassalle et al. showed that HCV infection was an independent predictor of angiographically documented CAD (adjusted OR = 4.2; 95% CI: 1.4–13.0) [9]. Similar results were reported in another study conducted in patients with CAD by Alyan et al. [22].

Moreover, in a study on hemodialysis patients, twenty-six nondiabetic, anti-hepatitis C virus- (HCV-) positive (15 females, mean age: 38 ± 8 years) and 26 anti-HCV-negative patients (15 females, mean age: 36 ± 5 years), all of whom had returned to PD or HD after renal transplant failure were studied to assess coronary flow reserve (CFR) by transthoracic Doppler echocardiography; HCV-positive patients showed lower CFR measurement than HCV-negative ones [23].

Maruyama et al. [24] had conducted a study on 217 consecutive cases of chronic HCV infection without overt heart disease and concluded that myocardial perfusion defects were found in 87% of the patients with chronic hepatitis C and improved with viral eradication with IFN therapy.

In contrast to our study, Völzke et al. [10] in their study conducted in the Pomeranian general population, Germany, enrolled 233 HCV Ab positive cases and 4033 control individuals and did not show an association between HCV seropositivity and cardiovascular diseases [10].

It is important to underline that those studies showing a positive association generally included a very large population of subjects, whereas the studies showing no association were generally conducted in smaller cohorts of patients at high risk for atherosclerosis.

The increased risk of CAD in HCV-infected persons may be related to a differential level of cytokines, which are markers of inflammation, thrombosis, and endothelial dysfunction; behavioral and social risk profile; malnutrition and/or inflammation pathway activation; or liver injury. More likely, a combination of these factors acts in concert to negate the protective effect of a favorable risk profile and increases the overall risk of CAD.

## 5. Conclusion

Finally we concluded that (1) HCV seropositive patients referred for coronary angiography were about 30.3% (which is greater than the prevalence of HCV seropositivity among general population in Egypt which was estimated to be about 14.7% and 18% among rural residents). (2) Patients proved to have CAD who are HCV Ab positive had more severe coronary lesions (estimated using Gensini score) than in seronegative one. (3) Patients proved to have CAD who are HCV Ab positive had a comparable prevalence of cardiovascular risk factors as seronegative patients except for diabetes and hypertension which are more prevalent in seronegative patients.

## Figures and Tables

**Table 1:** Demographic and clinical characteristics of the CAD positive and CAD negative groups.

Variable	Group 1 CAD positive (*n* = 344) *X* ± SD	Group 2 CAD negative (*n* = 165) *X* ± SD	*p* value	OR (95% CI)
Age (years)	53.37 ± 8.36	54.49 ± 9.93	0.38	

Gender				
(i) Male (%)	195 (56.7%)	108 (65.5%)	0.059	
(ii) Female (%)	149 (43.3%)	57 (34.5%)		

Risk factors				
(i) DM	175 (50.9%)	51 (30.9%)	<0.0001	2.32
(ii) HTN	230 (66.9%)	72 (43.6%)	<0.0001	2.6
(iii) Dyslipidemia	229 (66.6%)	84 (50.9%)	0.001	1.92
(iv) Smokers	153 (44.5%)	51 (30.9%)	0.003	1.79
(v) Positive family history	81 (23.5%)	27 (16.4%)	0.06	

Clinical presentation				
(i) Stress positive test (%)	15 (4.4%)	33 (20%)	<0.0001	
(ii) Unstable angina (%)	129 (37.5%)	126 (76.4%)		
(iii) NSTEMI (%)	76 (22.1%)	0 (0%)		
(iv) STEMI (%)	124 (36%)	6 (3.6%)		

Prevalence of HCV	118 (34.3%)	36 (21.8%)	0.004	1.87

CAD = coronary artery disease, NSTEMI = non-ST segment elevation myocardial infarction, STEMI = ST segment elevation myocardial infarction, *n* = number, *X* = mean, SD = standard deviation, OR = odds ratio, and CI = confidence interval.

**Table 2:** Demographic and clinical characteristics of the HCV-positive and HCV-negative groups.

Variable	Group 1A HCV positive (*n* = 118) *X* ± SD	Group 1B HCV negative (*n* = 226) *X* ± SD	*p* value
Age (years)	54.08 ± 8.37	53.52 ± 8.37	0.556

Gender			
(i) Male (%)	66 (55.9%)	129 (57.1%)	0.838
(ii) Female (%)	52 (44.1%)	97 (42.9%)	

Risk factors			
(i) DM	41 (34.7%)	134 (59.3%)	<0.0001
(ii) HTN	63 (53.4%)	167 (73.9%)	<0.0001
(iii) Dyslipidemia	80 (67.8%)	149 (65.9%)	0.727
(iv) Smokers	45 (38.1%)	108 (47.8%)	0.087
(v) Positive family history	29 (24.6%)	52 (23%)	0.745

CAD = coronary artery disease, *n* = number, *X* = mean, SD = standard deviation, DM = diabetes mellitus, HTN = hypertension, and EF = ejection fraction.

**Table 3:** Clinical presentation and echocardiographic and angiographic characteristics of HCV positive and HCV negative groups.

Variable	Group 1A HCV positive (*n* = 118) *X* ± SD	Group 1B HCV negative (*n* = 226) *X* ± SD	*p* value
Clinical presentation			
(i) Stress positive test (%)	5 (4.2%)	10 (4.4%)	0.984
(ii) Unstable angina (%)	45 (38.1%)	84 (37.2%)	
(iii) NSTEMI (%)	27 (22.9%)	49 (21.7%)	
(iv) STEMI (%)	41 (34.7%)	83 (36.7%)	

Echocardiographic data			
EF (%)	55.32 ± 10.31	55.24 ± 9.85	0.95

Angiographic data			
Gensini score	65.78 ± 7.81	57.42 ± 32.14	0.01

NSTEMI = non-ST segment elevation myocardial infarction, STEMI = ST segment elevation myocardial infarction, *n* = number, *X* = mean, SD = standard deviation, and EF = ejection fraction.
